# Antiemetic activity of abietic acid possibly through the 5HT_3_ and muscarinic receptors interaction pathways

**DOI:** 10.1038/s41598-024-57173-0

**Published:** 2024-03-19

**Authors:** Rubel Hasan, Abdulrahman Alshammari, Norah A. Albekairi, Md. Shimul Bhuia, Meher Afroz, Raihan Chowdhury, Muhammad Ali Khan, Siddique Akber Ansari, Irfan Aamer Ansari, Mohammad S. Mubarak, Muhammad Torequl Islam

**Affiliations:** 1 Department of Pharmacy, Bangabandhu Sheikh Mujibur Rahman Science and Technology University, Gopalganj, 8100 Bangladesh; 2 Department of Pharmacology and Toxicology, College of Pharmacy, King Saud University, Post Box 2455, 11451 Riyadh, Saudi Arabia; 3 BioLuster Research Center, Gopalganj, Dhaka, 8100 Bangladesh; 4 Department of Pharmaceutical Chemistry, College of Pharmacy, King Saud University, 11451 Riyadh, Saudi Arabia; 5 Department of Drug Science and Technology, University of Turin, 10124 Turin, Italy; 6 Department of Chemistry, The University of Jordan, Amman, 11942 Jordan; 7 Department of Chemistry, Indiana University, Bloomington, IN 47405 USA; 8 Pharmacy Discipline, Khulna University, Khulna, 9208 Bangladesh

**Keywords:** Emesis, Vomiting, *Gallus gallus domesticus*, Abietic acid, Molecular docking, Drug discovery, Diseases, Gastroenterology

## Abstract

The present study was designed to evaluate the antiemetic activity of abietic acid (AA) using in vivo and in silico studies. To assess the effect, doses of 50 mg/kg b.w. copper sulfate (CuSO_4_⋅5H_2_O) were given orally to 2-day-old chicks. The test compound (AA) was given orally at two doses of 20 and 40 mg/kg b.w. On the other hand, aprepitant (16 mg/kg), domperidone (6 mg/kg), diphenhydramine (10 mg/kg), hyoscine (21 mg/kg), and ondansetron (5 mg/kg) were administered orally as positive controls (PCs). The vehicle was used as a control group. Combination therapies with the referral drugs were also given to three separate groups of animals to see the synergistic and antagonizing activity of the test compound. Molecular docking and visualization of ligand-receptor interaction were performed using different computational tools against various emesis-inducing receptors (D_2_, D_3_, 5HT_3_, H_1_, and M_1_–M_5_). Furthermore, the pharmacokinetics and toxicity properties of the selected ligands were predicted by using the SwissADME and Protox-II online servers. Findings indicated that AA dose-dependently enhances the latency of emetic retching and reduces the number of retching compared to the vehicle group. Among the different treatments, animals treated with AA (40 mg/kg) exhibited the highest latency (98 ± 2.44 s) and reduced the number of retching (11.66 ± 2.52 times) compared to the control groups. Additionally, the molecular docking study indicated that AA exhibits the highest binding affinity (− 10.2 kcal/mol) toward the M_4_ receptors and an elevated binding affinity toward the receptors 5HT_3_ (− 8.1 kcal/mol), M_1_ (− 7.7 kcal/mol), M_2_ (− 8.7 kcal/mol), and H_1_ (− 8.5 kcal/mol) than the referral ligands. Taken together, our study suggests that AA has potent antiemetic effects by interacting with the 5TH_3_ and muscarinic receptor interaction pathways. However, additional extensive pre-clinical and clinical studies are required to evaluate the efficacy and toxicity of AA.

## Introduction

Emesis, or vomiting, is a typically unpleasant condition where stomach contents are forcefully expelled through the mouth. It is closely associated with the movement of the gastrointestinal system^1^. Vomiting is induced by toxins in the gut lumen or by irritation of the stomach through the gastrointestinal tract’s (GIT) mucosal chemoreceptors^2,3^. Besides ingesting toxins or irritants, several conditions, including food poisoning, gastroenteritis (diarrhea), motion sickness, hangovers, head injuries, intestinal obstruction, appendicitis, post-operative factors, and elevated intracranial pressure, can also lead to vomiting and nausea^4^. In addition, several adverse reactions to radiation therapy and cancer treatment can cause emesis^5^. There is evidence that emesis and GI disturbances can be induced by microbes and their secretions^6–8^. However, emesis is a very complex process, initiated by sending emetogenic stimuli to the vomiting center (VC) in the medulla oblongata^9^. Several important parts, such as the chemoreceptor trigger zone (CTZ), which is situated in the region postrema on the floor of the fourth ventricle and activated by certain blood-borne poisons or medications, play a crucial role in inducing emesis^10^. Vomiting triggered by the CTZ begins when its receptors detect emetogenic toxins in the blood and cerebrospinal fluid (CSF) and relay this information to the neighboring nucleus tractus solitarius (NTS). Abdominal vagal afferents that identify potentially emetogenic substances (e.g., uremic toxins, apomorphine, cardiac glycosides, and chemotherapeutic agents) in the lumen also terminate here^11^. The other sites besides the CTZ that relay information to the VC to induce emesis include the GI tract (stimulated by toxins and food consumption), the vestibular system, and the higher centers in the cortex and thalamus^12^. Electrical stimulation of all of these structures can induce emesis^13^. A vomiting stomach releases bicarbonate into the body and HCl into the gastric lumen. During vomiting, the body expels HCl while accumulating bicarbonate^14^. At the onset of vomiting, the lower esophageal sphincter relaxes, the stomach contracts intrinsically, and the vomit passes from the stomach into the esophagus. The abdominal and inspiratory muscles then contract, forcing the vomit to be expelled into the mouth^15^. The VC carries histamine (H_1_), neurokinin type 1 (NK_1_), serotonin 2 (5HT_2_), and muscarinic receptors, while dopamine (D_2_), μ (mu)-opioid, and serotonin 3 (5HT_3_) receptors are prevalent in the CTZ. In addition, after activating the receptor, 5HT_3_ has a peripheral action in the GIT, along with 5HT_4_ and D_2_^16^. In this regard, serotonin (5HT) receptors have been linked with vagal afferent and peripheral neural pathways^17^. They are stimulated by different stimuli and are responsible for the emetic process^18,19^. Several types of adrenergic (α_2_), CB_1_, and GABA_B_ receptors are also liable for inducing emesis^20,21^.

At present, various antiemetic drugs are used to treat nausea and vomiting. These can be classified as 5HT antagonists, anti-dopaminergic drugs, antihistamines, anticholinergic drugs, NK_1_-receptor inhibitors, corticosteroids, and cannabinoids^22^. Prolonged use of these medications is associated with unfavorable consequences, such as spasms, convulsions, or muscle weakness^23,24^. Therefore, the search for new and safe medications is the demand of time. Natural products have thus become indispensable in the current treatment approach because of their minimal adverse reactions and practical benefits^25^. The exploration of novel antiemetic drugs that are obtained from natural sources continues to focus on mechanism-based methods that involve specific molecular and cellular targets. Alkaloids, flavonoids, glucosides, cannabinoids, hydroxycinnamic acids, polysaccharides, diarylheptanoids, phenylpropanoids, terpenes, and saponins are used for finding potential new antiemetic medication drugs^26^.

Abietic acid (1R, 4aR, 4bR, 10aR)-1,4a-dimethyl-7-(propan-2-yl)-1,2,3,4,4a,4b,5,6,10,10a-octahydrophenanthrene-1-carboxylic acid) is a diterpenoid acid that is found in the resin of some coniferous plants, including pine and spruce. Abietic acid (AA) has different therapeutic activities, including antiviral, antibiotic, antifungal, anticancer, neuroprotective (Alzheimer’s disease), and antioxidant activities^27^. Moreover, AA prevents stomach secretions, indicating that it might be used as an antiulcer medication^28^, and has a cytotoxic effect^29^. In a study conducted by Fernández and coworkers, it was shown that AA possesses potent in vivo anti-inflammatory activity via topical and oral treatment^30^. There are several in vivo and in vitro models available for assessing the antiemetic activities of a compound or plant extract, one such model is the chick emesis^31^. In this study, oral administration of copper sulfate (CuSO_4_⋅5H_2_O) causes emesis in young chickens (*Gallus gallus domesticus*). The standard test sample is administered orally 30 min before CuSO_4_⋅5H_2_O. Evaluation of the antiemetic activity of the test sample is achieved by contrasting the number of retches with control groups^32^. On the other hand, the drug research and development process can be sped up and kept less expensive by using the computational drug discovery method. The diversity of data on biological macromolecules has significantly increased, and as a result, computational drug discovery is currently applied to nearly all stages of the process of finding and developing drugs. Additionally, it enables the prediction of pharmacokinetics and binding sites, both of which are vital in determining the mechanistic stages and binding when identifying and developing prospective drug candidates^33–35^. Accordingly, this study aimed to examine the antiemetic effects of AA on copper sulfate-induced emesis in young chickens. Simultaneously, a computational analysis was performed to investigate molecular interactions that may be liable for the observed effect, as well as to assess the pharmacokinetic and toxicological properties of AA.

## Materials and methods

### Chemical reagents and standards

AA (CAS No. 514-10-3) was purchased from Sigma-Aldrich (USA), while copper sulfate pentahydrate (CuSO_4_⋅5H_2_O) was obtained from Merck (India). Reference drugs, ondansetron (OND), domperidone (DOM), hyoscine butyl bromide (HYS), aprepitant (APT), and diphenhydramine (DHM) were purchased from Incepta, Beximco, Opsonin, Beacon, and Eskayef Pharma Ltd., Bangladesh, respectively.

### Selection and preparation of test and control groups

Based on a review of the literature, we selected two concentrations of the test sample (lower and higher). We prepared the sample’s mother solution at a concentration of 50 mg/kg by dissolving it in distilled water (DW) and a small amount of Tween 80 (0.5%) used as a co-solvent. The mother solution was then diluted at concentrations of 20 and 40 mg/kg. In contrast, doses of the referral drugs were chosen by converting human doses to animal doses supported by animal dose calculation protocol and literature procedures^8,36^. The reference drug’s solutions were also prepared by thoroughly mixing them into DW (where a small amount of Tween 80 was used as a co-solvent) at concentrations of 16, 6, 10, 21, and 5 mg/kg for the drugs APT, DOM, DHM, HYS and OND, respectively. Three combined doses of AA (40 mg/kg), DOM, HYS, and OND were also prepared for the co-treatments.

### Experimental animals

Young chicks (*Gallus gallus domesticus*) of both genders, with a weight range of 40–45 g, 2 days old, were purchased from Provita Feed and Hatcheries Ltd. at Road-3, House-270, Baridhara DOHS, Dhaka Division, 1206 Bangladesh. All chicks were kept at the pharmacology lab of Bangabandhu Sheikh Mujibur Rahman Science and Technology University, Gopalganj, for the present study. The chicks were given free access to regular food and water. They were maintained at 27 ± 2 °C with a 12-h dark/light cycle under controlled illumination before the test started. After 12 h of fasting, the antiemetic test was carried out. This study was approved by the Department of Pharmacy and the Ethical Committee of Bangabandhu Sheikh Mujibur Rahman Science and Technology University (#bsmrstu-phr-17PHR049-01). In addition, all methods were carried out in accordance with relevant guidelines and regulations, and all methods were reported in accordance with ARRIVE guidelines (https://arriveguidelines.org).

### In vivo protocol

The procedures outlined by Akita et al.^32^ were used to conduct the study with a few minor modifications. The chicks were divided into eleven groups of six each. Before receiving the treatments, each bird was retained in a sizable, clear plastic container for 10 min. Using DW, the test sample (AA) was prepared in two different doses (20 and 40 mg/kg), which were then administered orally. The reference drugs APT, DOM, DHM, HYS, and OND were orally given at doses of 16, 6, 10, 21, and 5 mg/kg b.w. The lower dose (20 mg/kg) of AA did not exhibit any significant synergistic or antagonistic effects in the combination therapy; therefore, the three reference medicines, DOM, HYS, and OND, were combined with AA at a dose of 40 mg/kg. Then, animals were orally given these combined doses to assess their synergistic or antagonistic effects. The other two referral drugs were omitted for combination therapy due to their inadequate antiemetic properties when given alone to the animals. DW with a small amount of tween 80 (0.5%) was used as a control group (vehicle). It was given orally at a dose of 150 mL/kg b.w. Each chick specimen had a 30-min treatment period before having emesis caused by the oral gavage of CuSO_4_⋅5H_2_O at a dose of 50 mg/kg of b.w. The latency period is the duration of time between the administration of the CuSO_4_⋅5H_2_O treatment and the occurrence of the first retch, then, the total number of retches within 10 min of receiving CuSO_4_⋅5H_2_O treatment and the latency were carefully noted. Compared to the vehicle group, we calculated the percentage reduction in retches and prolongation in latency according to the following formula:

$$\text{\% increase in latency}=\frac{{\text{M}}-{\text{N}}}{{\text{M}}}\times 100,$$

$$\text{\% decrease in retches}=\frac{{\text{C}}-{\text{D}}}{{\text{C}}}\times 100,$$

where M: is the mean of latency in seconds in standard and test groups, N: is the mean of latency in seconds in the vehicle group, C: is the mean of retches in the vehicle group, and D: the mean of retches in the standard and test groups.

### Statistical analysis

The values of the antiemetic efficacy are expressed as the mean and standard error of the mean (SEM). The Graph Pad Prism (version 6.0) is a statistical computer application that was used to estimate the variations’ statistical significance which was determined at a 95% confidence limit. p values of < 0.05 are considered significant, whereas p values of p < 0.0001 are very significant.

### In silico analysis

#### Homology model and preparation of receptors

Based on published research, we selected nine receptors to perform molecular docking and ligand-receptor visualization. We developed a homology model because the human 5HT_3_ receptor’s 3D structure wasn’t available in the RCSB Protein Data Bank^37^. Human 5HT_3_ receptor homology modeling was achieved using the SWISS-MODEL^38^. The UniProt database (http://www.uniprot.org) was used to retrieve the protein’s sequence, and the NCBI BLAST program was employed to conduct a BLAST analysis to determine the best template^39^. The GMQE^40^ and a Ramachandran plot using ProCheck^41^ methods were used to evaluate the 5HT_3_ homology modeling structures. D_2_ (PDB ID: 6LUQ)^42^, D_3_ (PDB ID: 3PBL)^43^, H_1_ (PDB ID: 3RZE)^44^, M_1_ (PDB ID: 6WJC), M_2_ (PDB ID: 5ZK8), M_3_ (PDB ID: 4U15), M_4_ (PDB ID: 7V6A), M_5_ (PDB ID: 6OL9)^45^, and NK_1_ (PDB ID: 6HLO)^46^ were obtained from the RCSB Protein Data Bank (https://www.rcsb.org/). After collecting receptors, the PyMol software program (v2.4.1) was used to remove any extraneous molecules, such as lipids, heteroatoms, and water molecules, from the protein sequence to optimize the receptors and prevent docking interference. Finally, using the SwissPDB Viewer software program and the GROMOS96 force field, the receptors’ shape and energy were optimized. The PDB file was then saved for use in molecular docking.

#### Collection and preparation of ligands

**Figure 1:**
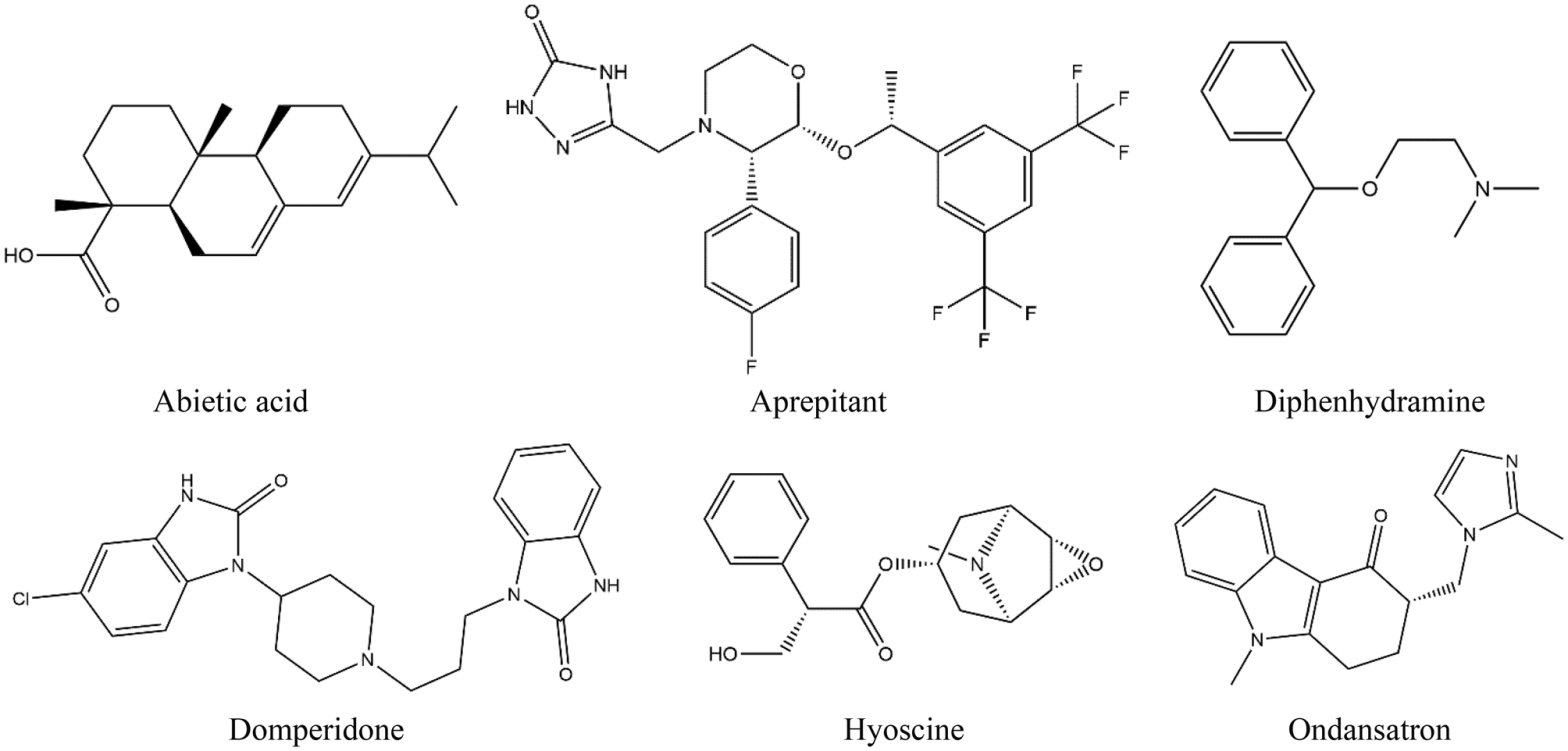
Chemical structures of abietic acid and reference drugs.

Based on the literature, we chose several well-known and commercially available antiemetic medications as reference ligands to compare the binding energy and molecular interaction with our test ligand (AA), with the focus on different emesis-causing receptors, to understand the root cause of the antiemetic mechanism. Afterward, the following were collected using the PubChem chemical database in SDF format (https://pubchem.ncbi.nlm.nih.gov/): several receptors, molecular docking, and prediction of pharmacokinetic features of the 3D conformers of abietic acid (Compound CID: 10569), aprepitant (Compound CID: 135413536), diphenhydramine (Compound CID: 3100), domperidone (Compound CID: 3151), hyoscine (Compound CID: 3000322), and ondansetron (Compound CID: 4595). Then, using the Chem3D 16.0 computer application, which is used for performing molecular docking and anticipating pharmacokinetics, the 3D conformers of the chemical agents were minimized, stored as SDF files, and transformed into MOL files, respectively. Finally, using the Gaussian View program (v5.0), all the ligands were optimized. Displayed in Fig. 1 are the chemical structures of AA and standard drugs.

#### Molecular docking study

Molecular docking was conducted using the PyRx software tool to predict the active binding energy of the drugs toward the active sites of receptors. For successful docking, the grid box dimensions were set at 85 × 80 × 75 Å along the x-, y-, and z-axes, respectively, and the calculation required 2000 steps^47,48^. The docking potential result is saved in ‘CSV’ format, and the ligand–protein complex is collected in PDB format to collect the ligand in PDBQT format. The interactions between ligand-receptors and the receptor’s active site were seen using the computer programs PyMol (v2.4.1) and Discovery Studio Visualizer (v21.1.020298). Then, the types of bonds, the number and length of hydrogen bonds, and each ligand-receptor interaction’s amino acid residues are documented.

#### Prediction of drug-likeness and pharmacokinetics

Drug-likeness is a qualitative assessment used to evaluate a molecule’s potential to be discovered and developed into an orally administered drug. A structural or physicochemical investigation was conducted to show similarities between the compounds and existing medications that were advanced enough in the research phase to be considered potential treatment options^49^. A chemical agent’s pharmacokinetics and drug-likeness may be calculated using a variety of web servers and applications. In this investigation, with the help of SwissADME, we discussed numerous criteria for evaluating the physicochemical characteristics of the test compound (http://www.swissadme.ch/index.php).

#### Toxicity prediction

To predict various toxicity parameters of any compound, ProTox-II online servers can be used. The ProTox-II web server is used to assess the safety profile of a chemical or compound by analyzing multiple toxicity endpoints, for instance, hepatotoxicity, carcinogenicity, mutagenicity, acute toxicity, immunogenicity, and cytotoxicity^50^. To evaluate the toxicity parameters, the Canonical SMILES were entered into the ProTox-II server (http://tox.charite.de/protox_II), which was collected from PubChem. The toxicity parameters of the selected compounds are listed in Table 1.

**Table 1:** Different treatments and their doses were investigated in animals.

Treatment groups	Composition	Dose (mg/kg)	Target receptor
Gr-I	Vehicle (0.5% Tween 80 dissolved in DW)	150 mL/kg	–
Gr-II	Abietic acid (AA)	20	Under investigation
Gr-III		40	
Gr-IV	Aprepitant (APT)	16	NK_1_
Gr-V	Domperidone (DOM)	6	D_2_
Gr-VI	Diphenhydramine (DHM)	10	H_1_
Gr-VII	Hyoscine butylbromide (HYS)	21	M_1_-M_5_ muscarinic acetylcholine
Gr-VIII	Ondansetron (OND)	5	5HT_3_
Gr-IX	DOM + AA-40	40 + 6	Under investigation
Gr-X	HYS + AA-40	40 + 21	Under investigation
Gr-XI	OND + AA-40	40 + 5	Under investigation

## Results

### In vivo investigation

**Figure 2:**
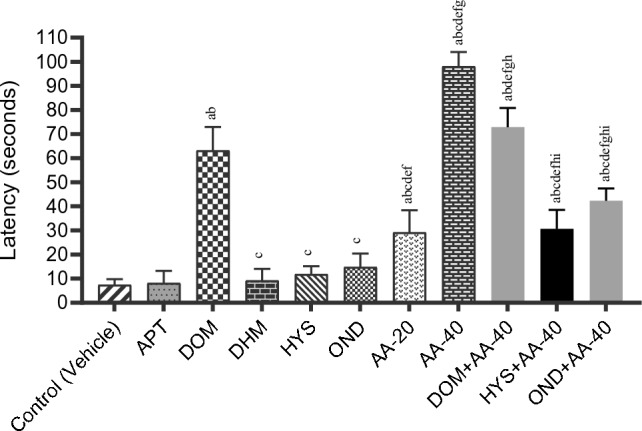
Latency observed in test samples, controls, and combinations [Values are the mean ± standard error of the mean (S.E.M.) (n = 6)]. ^a^Compared to the control (vehicle), ^b^compared to the APT; ^c^compared to the DOM; ^d^compared to the DHM; ^e^compared to the HYS; ^f^compared to the OND; ^g^compared to the AA-20; ^h^compared to the AA-40; ^i^compared to the DOM + AA-40; p < 0.05 (OND vs AA-20, AA-20 vs AA-40 + OND); p < 0.01 (OND Vs AA-40 + HYS); p < 0.001 (HYS vs AA-20, HYS vs AA-40 + HYS); p < 0.0001 (Vehicle vs DOM, vehicle vs AA-20, vehicle vs AA-40, vehicle vs AA-40 + DOM, vehicle vs AA-40 + HYS, vehicle vs AA-40 + OND, APT vs DOM, APT vs AA-20, APT vs AA-40, APT vs AA-40 + DOM, APT vs AA-40 + HYS, APT vs AA-40 + OND, DOM vs DHM, DOM vs HYS, DOM vs OND, DOM vs AA-20, DOM vs AA-40, DOM vs AA-40 + HYS, DOM vs AA-40 + OND, DHM vs AA-20, DHM vs AA-40, DHM vs AA-40 + DOM, DHM vs AA-40 + HYS, DHM vs AA-40 OND, HYS vs AA-40, HYS vs AA-40 + DOM, HYS vs AA-40 OND, OND vs AA-40, OND vs AA-40 + DOM, OND vs AA-40 + OND, AA-20 vs AA-40, AA-20 vs AA-40 + DOM, AA-40 vs AA-40 + DOM, AA-40 vs AA-40 + HYS, AA-40 vs AA-40 + OND, AA-40 + DOM vs AA-40 + HYS, AA-40 + DOM vs AA-40 + OND).

In our experiment, animals in the control (vehicle) group exhibited their first retching at 7.50 ± 0.92 s, whereas animals in the reference groups showed an elevated latency compared to the control group. Animals given DOM showed the highest latency (63.16 ± 3.99 s) among the selected reference drugs in this test. Values of the onset of retching for other reference groups are 8.17 ± 2.05, 9.16 ± 1.98, 11.83 ± 1.37, and 14.83 ± 2.27 s for APT, DHM, HYS, and OND, respectively. On the other hand, animals in the test groups (AA) exhibited a significant dose-dependent elevation in latency compared to the control group. Animals belonging to the AA-40 group exhibited the highest latency (98.00 ± 2.44 s) among all the test groups, while the other test group (AA-20) revealed 29.16 ± 3.77 s. The combination therapies demonstrated that AA notably increased latency when the animals were co-treated with the reference drugs compared to the reference drugs alone. The latency of the DOM + AA-40, OND + AA-40, and HYS + AA-40 groups is 72.83 ± 3.25, 42.33 ± 2.09, and 30.66 ± 3.21 s, respectively. The latency obtained from all treatment groups is illustrated in Fig. 2.

**Figure 3:**
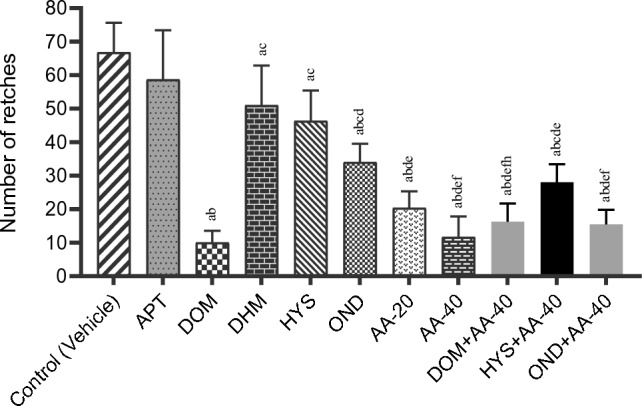
Number of retches observed in the test sample, controls, and combination [Values are mean ± standard error of the mean (SEM) (n = 6)]. ^a^Compared to the control (vehicle), ^b^compared to the APT; ^c^compared to the DOM; ^d^compared to the DHM; ^e^compared to the HYS; ^f^compared to the OND; ^g^compared to the AA-20; ^h^compared to the AA-40; p < 0.05 (Vehicle vs DHM, DOM vs AA-40 + HYS, DHM vs OND, OND vs AA-40 + DOM, AA-40 vs AA-40 + HYS); p < 0.01 (vehicle vs HYS, HYS vs AA-40 + HYS, OND vs AA-40 + OND); p < 0.001 (DOM vs OND, DHM vs AA-40 + HYS, OND vs AA-40); p < 0.0001 (vehicle vs DOM, vehicle vs OND, vehicle vs AA-20, vehicle vs AA-40, vehicle vs AA-40 + DOM, vehicle vs AA-40 + HYS, vehicle vs AA-40 + OND, APT vs DOM, APT vs OND, APT vs AA-20, APT vs AA-40, APT vs AA-40 + DOM, APT vs AA-40 + HYS, APT vs AA-40 + OND, DOM vs DHM, DOM vs HYS, DHM vs AA-20, DHM vs AA-40, DHM vs AA-40 + DOM, DHM vs AA-40 + OND, HYS vs AA-20, HYS vs AA-40, HYS vs AA-40 DOM, HYS vs AA-40 + OND).

The highest number of retches (66.83 ± 3.58) was noticed in the vehicle group in this test. A remarkable reduction in the number of retches in animals treated with reference drugs such as APT, DOM, DHM, HYS, and OND was observed as compared to the vehicle, where DOM exhibited the lowest (10.00 ± 1.46) retches among the selected reference drugs, even across all the treatment groups. The values of the number of retching for APT, DHM, HYS, and OND are 58.66 ± 6.03, 51.00 ± 4.87, 46.33 ± 3.70, and 34.00 ± 2.26, respectively. In the case of the test sample, there was a significant dose-dependent decrease in the number of retches, and the animals in the AA-20 and AA-40 groups displayed 20.33 ± 2.04 and 11.66 ± 2.52 retches, respectively. In the combination groups, the lowest number of retches exhibited was in the OND + AA-40 group (15.50 ± 1.76). The total number of retches for all treatment groups is shown in Fig. 3.

Animals belonging to the AA-20 and AA-40 groups showed an increase in percentage of latency compared to the vehicle group, which was 74.27 and 92.34%, respectively. Findings indicated that the latency period is elevated with the increase in doses in the test groups. However, animals treated with combined therapies also showed a significant elevation in the latency percentage; among the several combination treatments, DOM + AA-40 exhibited the highest latency percentage of 89.70%. In the case of a percentage decrease in retches, treatment with the test compound demonstrated a dose-dependent percentage decrease in retching. The highest percentage decrease in retching was observed in the DOM group (85.03%), though the drug’s combination therapy with AA showed a reduction in retching (75.56%). Our findings showed that the percentage decrease in retching for other combination groups are 58.10 and 76.80% for the HYS + AA-40 and OND + AA-40 groups, respectively. The percentage decrease in retching and the rise in the latency period for each treatment group are displayed in Table 2.

**Table 2:** Percentage increase in latency and reduction of retching in emetic animals of test and/or control groups.

Treatment groups	Decrease in retches (%)	Increase in latency (%)
Control (vehicle)	–	–
APT	12.22	8.20
DOM	85.03	88.12
DHM	23.68	18.12
HYS	30.67	36.60
OND	49.12	49.42
AA-20	69.57	74.27
AA-40	82.55	92.34
DOM + AA-40	75.56	89.70
HYS + AA-40	58.10	75.53
OND + AA-40	76.80	82.28

Control (vehicle): Distilled water (Dose:150 mL/kg); APT: Aprepitant (Dose:16 mg/kg); DOM: Domperidone (Dose: 6 mg/kg); DHM: Diphenhydramine (Dose: 10 mg/kg); HYS: Hyoscine (Dose: 21 mg/kg); OND: Ondansetron (Dose: 5 mg/kg); AA-20: Abietic Acid (Dose: 20 mg/kg); AA-40: Abietic Acid (Dose: 40 mg/kg); DOM + AA-40: Domperidone + Abietic Acid (Dose: 6 mg/kg + 40 mg/kg); HYS + AA-40: Hyoscine + Abietic acid (Dose: 21 mg/kg + 40 mg/kg) OND + AA-40: Ondansetron + Abietic Acid (Dose: 5 mg/kg + 40 mg/kg).

### In silico study

#### Homology modeling of human 5HT_3_ protein

**Figure 4:**
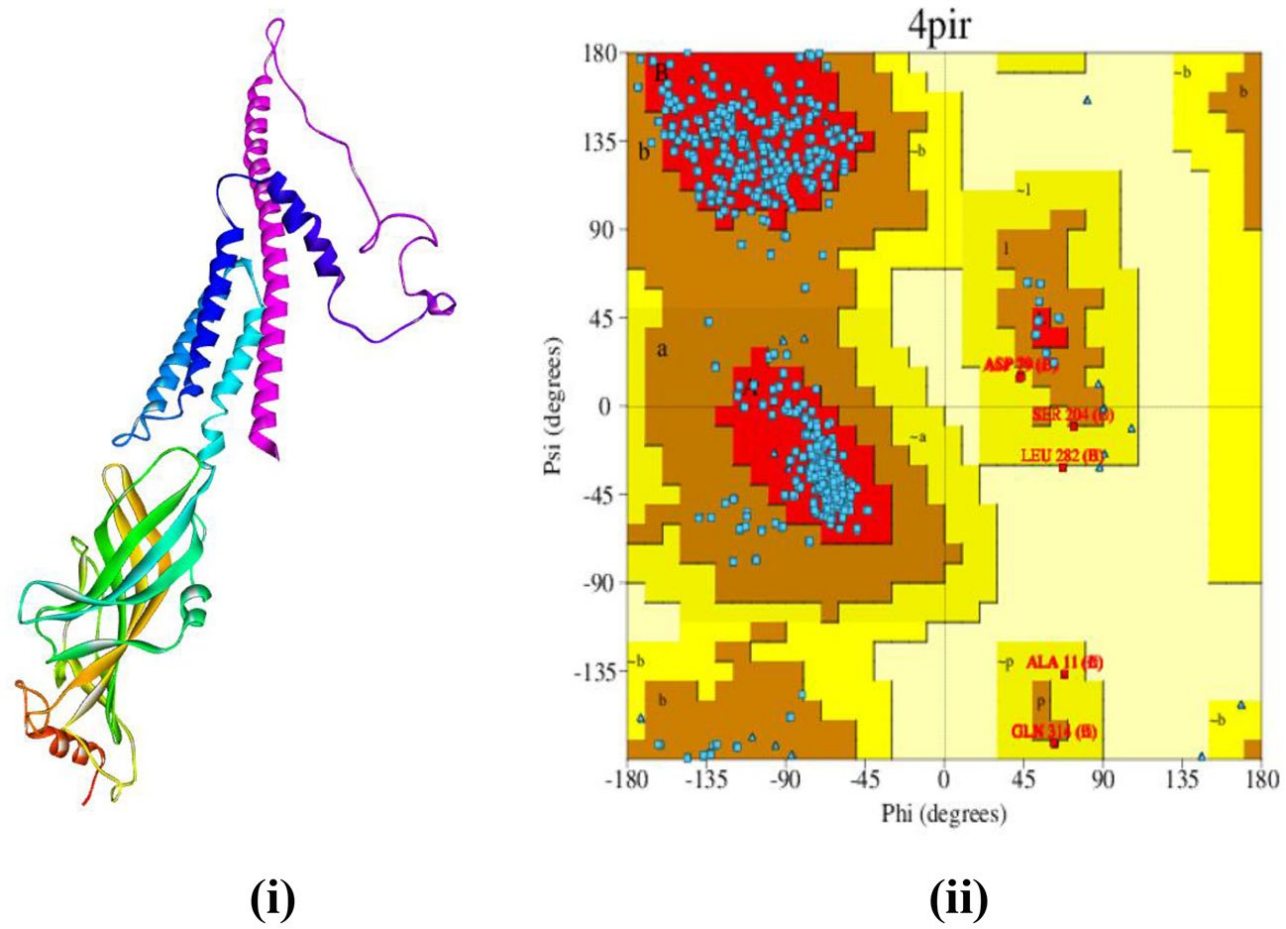
(**i**) The Swiss Model-built 3D structure of the human 5HT_3_ receptor, (**ii**) Ramachandran plot of the homology model 5HT_3_ protein for all non-glycine/proline residues.

Findings from the homology modeling show that the desired sequence and the template sequence of 4PIR (PDB ID), an X-ray crystallographic structure of the mouse 5HT_3_ receptor, have similar sequences. The target protein sequence shares 95% coverage and 86.95% identity with the template sequence, which also has a 58% sequence similarity. With a QMEAN of − 3.91 and a GMQE score of 0.72, the homology model of human 5HT_3_ was developed, suggesting high quality and consistency. To verify the accuracy and reliability of the residues’ Psi and Phi angles, the Ramachandran plot was designed. The plot revealed 1.81% Ramachandran outliers and 91.65% Ramachandran preferences in Fig. 4.

#### Molecular docking

A molecular docking approach was used to predict the possible binding energy between ligand and protein. Our in silico study revealed that the test ligand (AA) shows the highest docking score (− 10.2 kcal/mol) toward the M_4_ receptor among the selected emesis-inducing receptors, whereas the referral ligand HYS exhibited a reduced docking score for the test ligand against the same receptor. The test ligand also showed a higher docking score than HYS toward the other subtypes (M_1_, M_2_, and M_5_) of mAChRs except M_3_ (Table 3). AA also demonstrated higher binding affinity toward 5HT_3_ and H_1_ receptors, and the docking scores are − 8.1 and − 8.5 kcal/mol, respectively. While the selected referral ligands OND and DHM expressed binding affinity of − 6.9 and − 6.3 kcal/mol with the 5HT_3_ and H_1_ receptors, respectively, In the case of the dopamine receptor, the selected antagonist DOM elicited higher docking scores than AA toward its emesis-inducing subunits D_2_ and D_3_, and the values are − 9.6 and − 9.9 kcal/mol, respectively. This study also revealed that APT binds with the NK_1_ receptor by showing a remarkable binding interaction of − 12.7 kcal/mol, while AA exhibited a lower binding interaction of − 8.8 kcal/mol. The docking scores of all the drugs and test ligands used against the specified receptors are displayed in Table 3.

**Table 3:** Docking value (kcal/mol) of abietic acid and reference drugs against specified receptors liable for inducing emesis.

Ligands	Receptors										
	Common Name	5HT_3_	M_1_	M_2_	M_3_	M_4_	M_5_	H_1_	D_2_	D_3_	NK_1_
	PDB ID	–	6WJC	5ZK8	4U15	7V6A	6OL9	3RZE	6LUQ	3PBL	6HLO
DOM									− 9.6	− 9.9	
OND		− 6.9									
HYS			− 6.7	− 7.7	− 9.1	− 8.9	− 8.8				
APT											− 12.7
DHM								− 6.3			
AA		− 8.1	− 7.7	− 8.7	− 7.6	− 10.2	− 8.9	− 8.5	− 9	− 9.2	− 8.8

*DOM* domperidone, *OND* ondansetron, *HYS* hyoscine, *APT* aprepitant, *DHM* diphenhydramine, *AA* abietic acid.

#### Prediction of non-bond interactions between protein–ligand complexes

Findings from the in silico study demonstrated that ligands interact with receptors by establishing a variety of bonds, including hydrogen bonds (HB) (both conventional HB and carbon HB) and other types of bonds, including alkyl, pi-alkyl, sigma, pi-pi T-shaped, pi-sulfur, pi-cation, and pi-pi stacked bonds. For the 5HT_3_ receptor, AA showed a higher docking value of − 8.1 kcal/mol, while the standard drug OND revealed a docking value of − 6.9 kcal/mol. AA binds with the 5HT_3_ receptor by forming one hydrogen bond residue (HB), namely ILE98, in addition to showing several hydrophobic bonds (HP) with amino acid residues of PRO113, LYS25, PRO89, VAL95, and TYR114. In contrast, OND did not bind with the 5HT_3_ receptor through HBs but formed numerous numbers of HP bonds with specific amino acid residues of LEU260, LEU259, VAL237, LEU234, and VAL264. DOM exhibits strong antagonistic action against the D2 receptor with a docking score of − 9.6 kcal/mol by generating 4 HBs, namely THR433, SER430, HIS414, and ASP114. AA exhibited a docking value of − 9.2 kcal/mol and formed one HB, DOM also interacted with the D_3_ receptor by showing a higher docking value of − 9.9 kcal/mol with three HBs of VAL111, ASP110, and CYS181, whereas AA displayed one HBs with a certain amino acid residue of SER366 and a binding affinity of − 9.2 kcal/mol.

**Figure 5:**
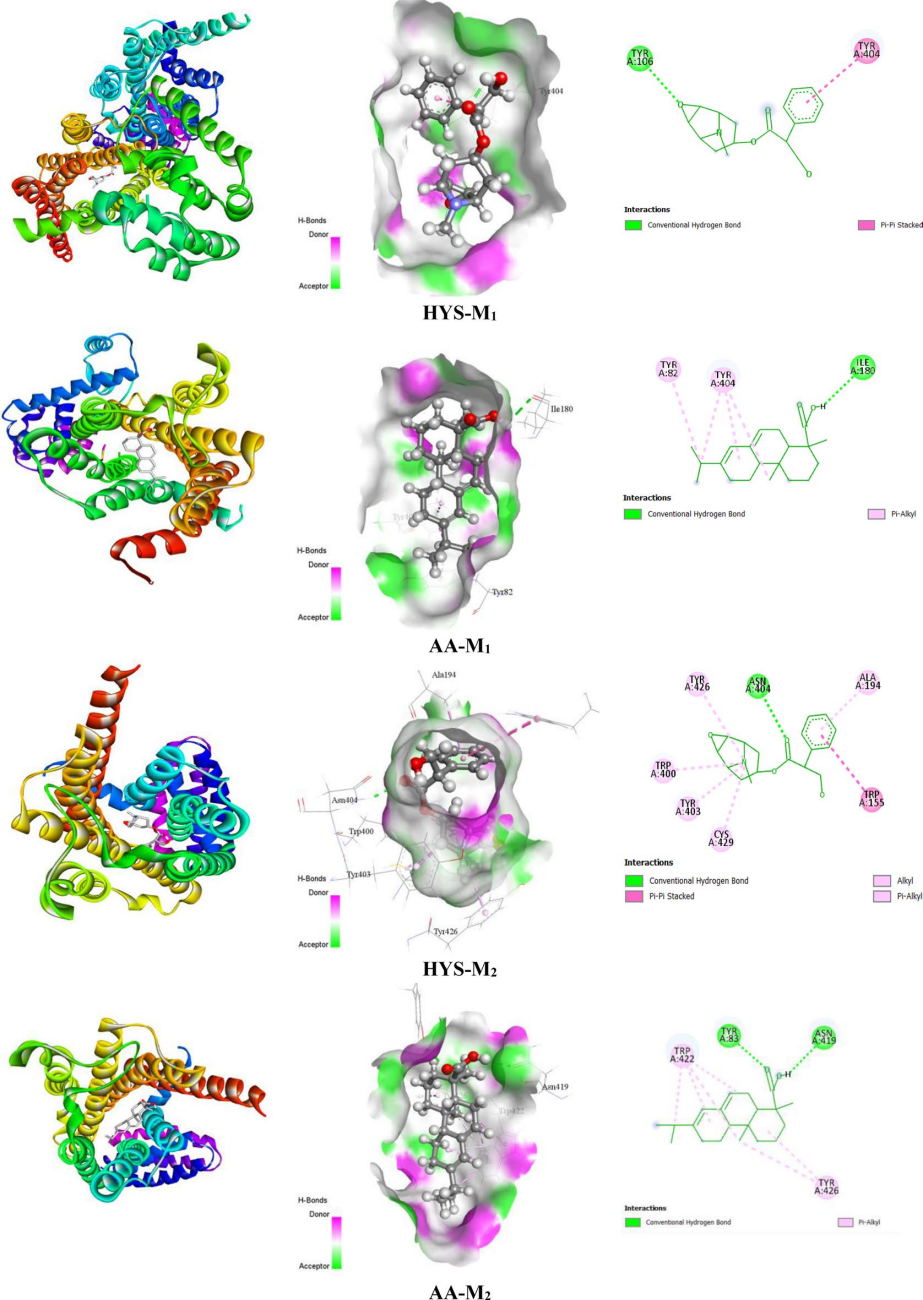

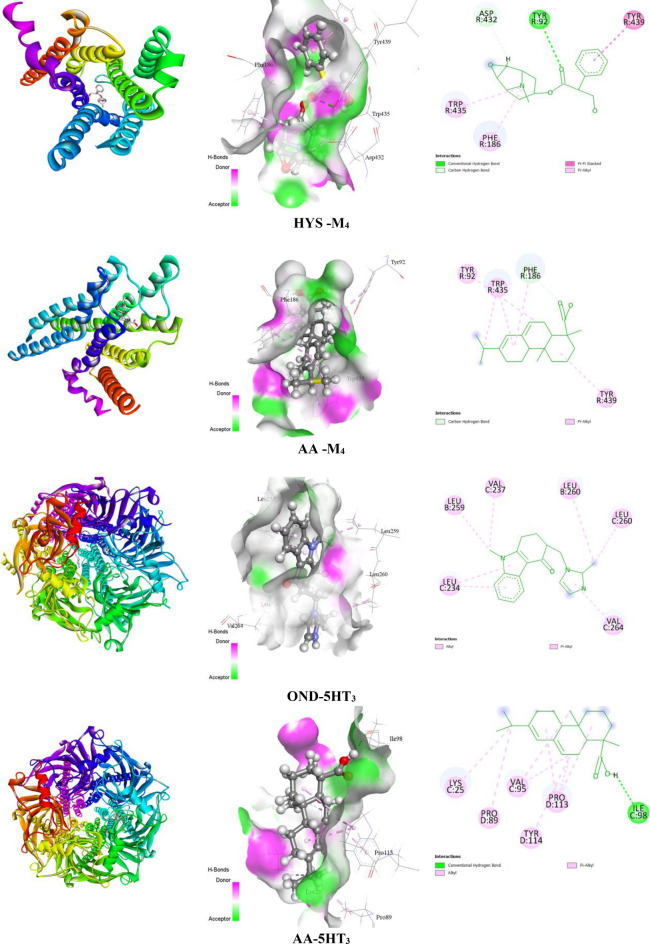
3D and 2D view of protein–ligand interaction and their binding sites with related amino acid residues.

Due to the interaction between the DHM and H_1_ receptor, which displayed a docking score of − 6.3 kcal/mol with no HBs, it formed several HP bonds, including particular amino acid residues of PHE116, PHE119, PRO202, ILE120, and ALA151. On the contrary, two HBs are formed, including ILE148 and SER68 amino acid residues, and they also obtained a greater binding energy of − 8.5 kcal/mol after docking AA with the H_1_ receptor. On the other hand, the binding scores of AA for the M_1_, M_2_, M_3_, M_4_, and M_5_ receptors were − 7.7, − 8.7, − 7.6, − 10.2, and − 8.9 kcal/mol, respectively. It is obvious that AA exhibited the highest binding affinity against the M_4_ receptor among all emesis-inducing receptors. Interaction is established between the AA and M_4_ receptors by the formation of one HB with a particular amino acid residue of PHE186 and four HP bonds, namely ASP432, TYR439, PHE186, and TRP435. In contrast, the standard drug HYS demonstrated docking values against M_1_, M_2_, M_3_, M_4_, and M_5_ receptors of − 6.7, − 7.7, − 9.1, − 8.9, and − 8.8 kcal/mol, respectively. However, two HBs are formed due to the interaction between the HYS and M_4_ receptors with the amino acid residues of TYR92 and ASP432. Additionally, HYS formed three HP bonds with specific amino acid residues of TYR439, PHE186, and TRP435. The highest level of docking value (− 12.7 kcal/mol) occurs from the interaction between the APT and NK_1_ receptor. It also revealed four HBs with the amino acid residues of ASN89, TRP184, GLN165, and HIS265. Furthermore, APT exhibited numerous numbers of HP bonds. Moreover, AA showed two HB namely HIS265 and THR201 and several HP bonds after binding with the NK_1_ receptor. The number of HBs, ligands, receptors’ bond types, HB lengths, amino acid residues, and the interacted ligand-receptor pockets are represented in Table 4 and Fig. 5.

**Table 4:** Amino acid residues, number of hydrogen bonds, and hydrogen bond length of non-bond interactions between the selected ligands and receptors.

Proteins	Ligands	No. of HB	HB residues	HB length (Å)	Other bond residues
5HT_3_	OND	0	–	–	LEU260, LEU259, VAL237, LEU234, VAL264
	AA	1	ILE98	2.20	PRO113, LYS25, PRO89, VAL95, TYR114
D_2_	DOM	4	THR433, SER430, HIS414, ASP114,	2.73, 2.02, 2.35, 2.13, 2.61	CYS118, PHE189, PHE410, VAL91, VAL115, LEU94
	AA	1	THR433	1.73	ILE184, VAL91, PHE110, PHE410, PHE411, TYR437
D_3_	DOM	3	VAL111, ASP110, CYS181,	2.70, 2.01, 2.97	VAL111, CYS114, PHE343, HIS349, PHE345, VAL107, ILE183, VAL189, VAL350, PHE106, LEU89, PHE346, VAL86, CYS181, PHE106
	AA	1	SER366	2.33	VAL86, LEU89, VAL107, ILE183, PHE106, TYR373
H_1_	DHM	0	–	–	PHE116, PHE119, PRO202, ILE120, ALA151
	AA	2	ILE148, SER68	2.55, 3.057	TRP152, VAL71, LEU149
M_1_	HYS	1	TYR106	2.83	TYR404
	AA	1	ILE180	2.47	TYR82, TYR404
M_2_	HYS	1	ASN404	2.38	TRP155, CYS429, ALA194, TRP400, TYR403, TYR426
	AA	2	TYR83, ASN419,	2.20, 2.37	TRP422, TYR426
M_3_	HYS	3	ASN507, ALA238, TYR529	2.08, 2.40, 2.82	TYR529, CYS532, TYR148, TRP503, TYR506, ALA235, VAL510
	AA	0	–		TYR127, PHE221
M_4_	HYS	2	TYR92, ASP432	2.87, 2.77	TYR439, PHE186, TRP435
	AA	1	PHE186	2.24	TYR92, TRP435, TYR439
M_5_	HYS	2	ASN459, ASP110	2.65, 2.80	TYR111, TRP455, TYR458, TYR481, ALA 201
	AA	2	HIS478, TRP477	2.76, 2.14	TRP477, VAL474
NK_1_	APT	4	ASN89, GLN165, TRP184, HIS265	2.86, 2.82, 2.26, 2.78	PHE268, HIS197, PRO112, ILE113, MET295, ILE204, MET291, ILE182, PHE264, TRP261
	AA	2	HIS265, THR201	2.70, 2.37	ILE113, HIS108, PHE264, PHE268,

*HB* hydrogen bond, *AA* abietic acid, *DOM* domperidone, *OND* ondansetron, *HYS* hyoscine, *DHM* diphenhydramine, *APT* aprepitant.

#### Estimation of in silico pharmacokinetics and drug-likeness (ADME)

**Figure 6:**
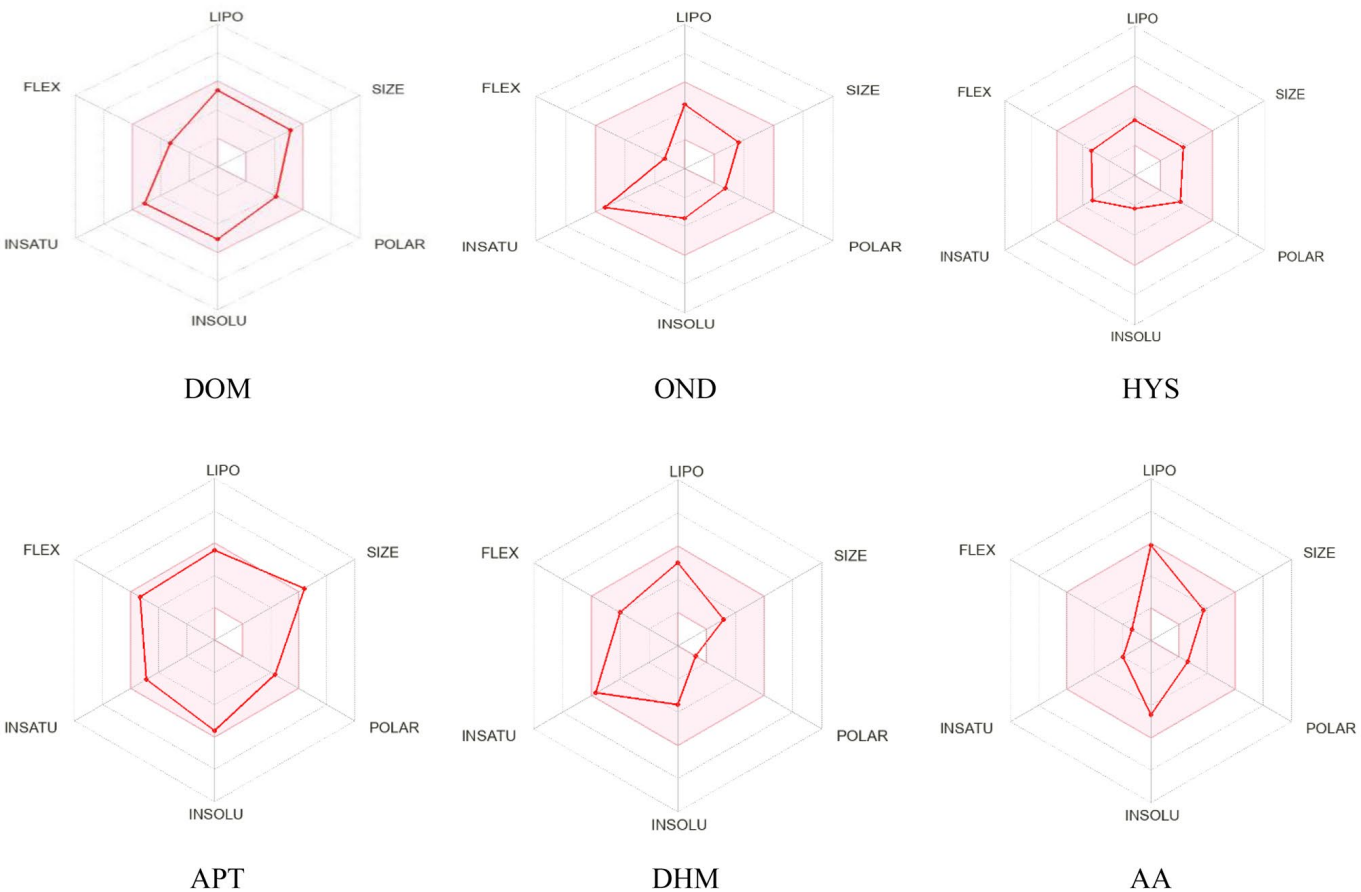
Summary of physiochemical, toxicological, and pharmacokinetics properties of selected compounds. [The colored zone is the suitable physicochemical space for oral bioavailability; SIZE: 150 g/mol < MV < 500 g/mol; INSOLU (Insolubility): − 6 < log S (ESOL) < 0; LIPO (Lipophilicity): − 7 < XLOGP3 <  + 5.0; INSATU (In saturation): 0.25 < Fraction Csp3 < 1; POLAR (Polarity): 20 Å^2^ < TPSA < 130 Å^2^; FLEX (Flexibility): 0 < num. rotatable bonds < 9].

Drug-likeness is an important characteristic of a drug candidate and involves developing a chemical substance into a medication and assessing its pharmacokinetics. The in silico ADMET method play key roles in drug discovery and development. A high-quality drug candidate should not only have sufficient efficacy against the therapeutic target, but also show appropriate ADMET properties at a therapeutic dose. Hydrogen bond donors (HBD), molecular weight (MW), hydrogen bond acceptor (HBA), molar refractivity (MR), and Log P are the primary parameters used to assess drug-likeness. According to the in silico ADMET results, all the drugs used have MW less than 500 Dalton without APT. According to Lipinski’s rule of five, a drug candidate must follow the values of HBD (≤ 5) and HBA (≤ 10) to be developed as a therapeutic, moreover, the APT contains 12 HBA, which breaks Lipinski’s rule of five. Results also demonstrated that HYS and OND are soluble in water, whereas others are comparatively soluble in water. Only APT is partially absorbed by GIT; other drugs are highly absorbed. Results also showed that AA has all the pharmacokinetics and physiochemical properties to be a drug-like compound. The compound also followed the Egan, Ghose, and Veber rules to assure drug-likeness but violates the Lipinski rules because its MLOGP is less than 4.15. Other parameters, for instance, P-gp substrate, TPSA, CYP2C19 inhibitor, BBB permeability, and bioavailability score of AA and reference drugs are given in Table 5 and a graphical representation in Fig. 6.

**Table 5:** The pharmacokinetics and physicochemical characteristics of Abietic acid and reference drugs are predicted by SwissADME.

Parameters	DOM	OND	HYS	APT	DHM	AA
Physicochemical properties						
MF	C_22_H_24_CIN_5_O_2_	C_18_H_19_N_3_O	C_17_H_21_NO_4_	C_23_H_21_F_7_N_4_O_3_	C_17_H_21_CINO	C_20_H_30_O_2_
MW	425.91	293.36	303.35	534.43	255.35	302.45
Number of heavy atoms	30	22	22	37	19	22
Number of aromatic heavy atom	18	14	6	17	12	0
HBA	3	2	5	12	2	2
HBD	2	0	1	2	0	1
TPSA (Å^2^)	78.82 Å^2^	39.82Å^2^	62.30 Å^2^	83.24 Å^2^	12.47 Å^2^	37.30 Å^2^
MR	124.08	87.39	83.48	118.82	79.10	92.22
Solubility						
Solubility (water)	Moderately soluble	Soluble	Soluble	Moderately soluble	Moderately soluble	Moderately soluble
Lipophilicity						
log Po/w (XLOGP3)	3.90	2.29	0.98	4.20	3.27	4.78
Log P	3.28	1.75	1.19	4.05	3.16	4.54
Pharmacokinetics						
GI absorption	High	High	High	Low	High	High
BBB permeant	Yes	Yes	No	No	Yes	Yes
P-gp substrate	Yes	Yes	No	Yes	No	No
BIO Score	0.55	0.55	0.55	0.55	0.55	0.85
CYP2C19 int	Yes	Yes	No	No	No	Yes
Medicinal chemistry						
Synthetic accessibility	2.83	3.13	4.03	4.57	2.12	4.80
Drug likeness						
Lipinski	Yes; 0 violation	Yes; 0 violation	Yes; 0 violation	Yes; 1 violation: MW > 500	Yes; 0 violation	Yes; 1 violation: MLOGP > 4.15
Ghose	Yes	Yes	Yes	No; 2 violations: MW > 480, WLOGP > 5.6	Yes	Yes
Veber	Yes	Yes	Yes	Yes	Yes	Yes
Egan	Yes	Yes	Yes	No;1 violation: WLOGP > 5.88	Yes	Yes
Muegge	Yes	Yes	Yes	No;1 violation: H-acc > 10	Yes	Yes

*MF* molecular formula, *LogP* Log *P*_*o/w*_ (MLOGP) (optimum: ≤ 5), *MW* molecular weight (g/mol) (optimum: ≤ 500), *HBA* hydrogen bond acceptor, (optimum: ≤ 10), *MR* Molar refractivity (optimum: ≤ b140), *HBD* hydrogen bond donor (optimum: ≤ 5), *CYP2C19 int* CYP2C19 inhibitor, *TPSA* topological polar surface area, *BIO Score* bioavailability score, *DOM* domperidone, *OND* ondansetron, *HYS* hyoscine hydrobromide, *APT* aprepitant, *DHM* diphenhydramine, *AA* abietic acid.

#### In silico toxicity of the selected compounds

Toxicological assessment of small molecules is crucial in predicting their acceptability for use in animal and human models. The toxicity parameters of a drug candidate can be predicted using the online server Protox-II. According to our in silico toxicity assessment, DOM, HYS, and AA are categorized into toxicity class 4 (harmful if swallowed, 300 < LD50 ≤ 2000). On the other hand, OND and DHM fall into toxicity class 3 (toxic if swallowed, 50 < LD50 ≤ 300). In the case of organ (liver) toxicity, our findings predict that all the referral drugs are inactive, whereas AA expressed a positive result (active). Results of the risk assessments of the selected compound were also carried out by using toxicity end point estimation, where HYS, DHM, and AA exhibited no toxicity in the cases of carcinogenicity, immunotoxicity, mutagenicity, and cytotoxicity, but the prediction showed a positive result (active) for the drugs DOM and OND in the cases of immunotoxicity and mutagenicity, respectively, and the other mentioned toxicity parameters are inactive for these two drugs. The different toxicity parameters and their status or values for our selected chemical compounds are given in Table 6.

**Table 6:** Prediction of different toxicity parameters of Abietic acid and selected referral drugs using Protox-II online tools.

Properties	Parameters	DOM	OND	HYS	DHM	AA
Toxicity	LD_50_	715 mg/kg	95 mg/kg	1275 mg/kg	64 mg/kg	1000 mg/kg
	Toxicity class	4	3	4	3	4
	Hepatotoxicity	Inactive	Inactive	Inactive	Inactive	Active
	Carcinogenicity	Inactive	Inactive	Inactive	Inactive	Inactive
	Immunotoxicity	Active	Inactive	Inactive	Inactive	Inactive
	Mutagenicity	Inactive	Active	Inactive	Inactive	Inactive
	Cytotoxicity	Inactive	Inactive	Inactive	Inactive	Inactive

*DOM* domperidone, *OND* ondansetron, *HYS* hyoscine hydrobromide, *DHM* diphenhydramine, *AA* abietic acid.

## Discussion

Orally consumed poisonous CuSO_4_ can trigger a particular vagal-induced vomiting reaction and can injure the mucous membranes in the GIT since CuSO_4_ is effective as an oxidizing and corrosive agent^51,52^. The GIT’s visceral afferent nerve fibers are stimulated by peripheral processes, which subsequently transmit the stimulation toward the VC, causing the act of vomiting^53,54^. The principal mediator of emesis is a CTZ in the medulla that is located outside the blood-brain barrier (BBB). It works by triggering a second region of VC^55^. Once initiated, vomiting proceeds in two stages, including retching and ejection. A VC or a central pattern generator may be in the area postrema, and the nearby NTS controls the muscles that are responsible for that series of events^56^. In addition, emesis is caused by local neuronal release of 5HT in the area postrema, triggering different subtypes of 5HT such as 5HT_3_ and 5HT_4_ receptors^57^. Several other receptors, such as H_1_^58^, mAChRs (M_1_–M_5_)^59^, NK_1_^60^, and different subtypes (D_2_ and D_3_) of dopamine receptors are also involved in the emetic process^18^, which have a significant impact on stimulating CTZ for inducing emesis.

APT is a highly selective antagonist of the NK_1_ receptor used to manage and treat chemotherapy-induced and postoperative nausea and vomiting^61^. Our findings showed that APT exhibits a lower efficacy to reduce the emetic symptoms of the animals as the number of retches and onset of the retching period were comparatively close to those of the vehicle group. The referral drug DOM is widely used for the treatment of nausea and vomiting because it is a selective systemic antagonist of dopamine D_2_ and D_3_ receptors, which reduces the activity of these receptors at the CTZ in the brain to alleviate the emetic symptoms^62^. Our findings from the in vivo investigation demonstrated that the animals given DOM revealed 10.00 ± 1.46 retches, while the animals belonging to the vehicle group showed 66.83 ± 3.58 retches, indicating the drug’s notable emesis diminishing capability. Additionally, DOM remarkably elevated the latency period (63.16 ± 3.99 s) compared to the control group (7.5 ± 0.92 s), which is also evidence of the drug’s remarkable antiemetic properties. In this context, histamine plays an essential role in sending signals from the GI system related to food allergies and histamine seafood poisoning to the brain, leading to vomiting^63,64^. Antihistamine drugs such as DHM play an important role in minimizing the emetic process by antagonizing the H_1_ receptor^23,65^. In our study, DHM-treated animals exhibited comparatively lower efficacy than other treatment groups and failed to manage CuSO_4_⋅5H_2_O-mediated emesis. In the case of the 5HT_3_ receptors, they are implicated in the process of causing vomiting through interpreting information from the digestive system. These receptors significantly influence the enteric nervous system’s ability to control bowel movements and peristalsis^66^. 5HT_3_ antagonists like OND hinder the activity of the receptor and alleviate vomiting. In this in vivo test, the OND and HYS-treated animal groups reduced the number of retches compared to the vehicle group to 34.00 ± 2.26 and 46.33 ± 3.70, respectively. Furthermore, the OND and HYS-ingested groups also showed an elevated latency period of 14.83 ± 2.27 and 11.83 ± 1.37 s, respectively. All these findings indicate the potent antiemetic features of drugs. In this respect, the test compound (AA) also has significant capability to alleviate the emetic condition, as the animals treated with AA demonstrated an incredibly reduced number of retches and an elevation in the onset of retching. These results show that AA exhibits better antiemetic activity than the referral drugs APT, DHM, HYS, and OND to mitigate CuSO_4_⋅5H_2_O induced-emesis in the in vivo experiment, as animals given AA expressed a lower number of retching and an elevated onset period. In addition, findings revealed that AA shows a dose-dependent antiemetic response. The higher dose of the test compound showed longer latency (98.00 ± 2.44) than the referral drug DOM, and the number of retching was also comparatively similar (10 ± 1.46 and 11.66 ± 2.52 for DOM and AA-40, respectively). These findings indicate remarkable potency compared to DOM to mitigate the emetic process.

**Figure 7:**
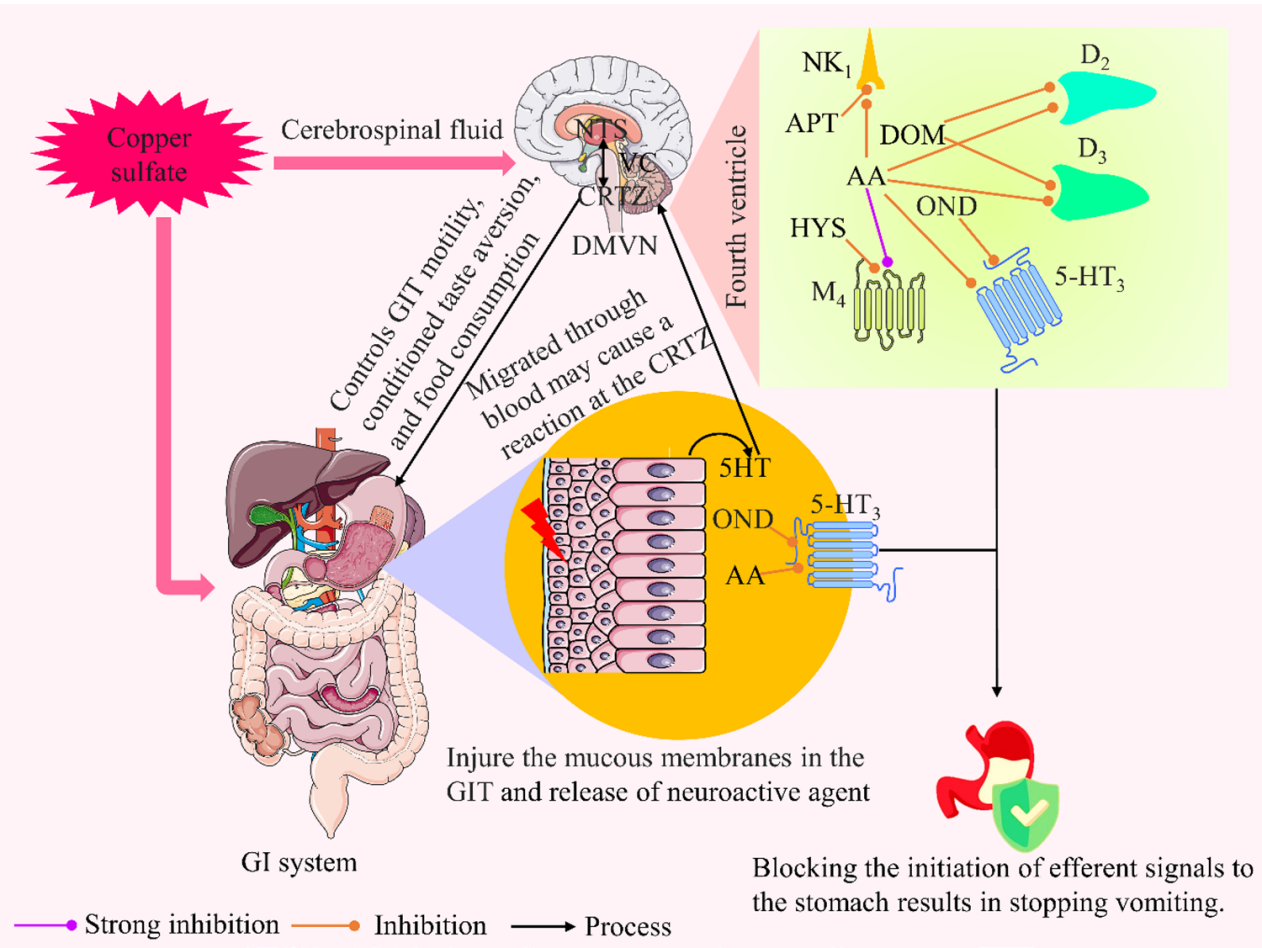
The suggested anti-emetic mechanism of the test compound (abietic acid) compared to the selected standard drugs. [This Fig illustrates the anti-emetic mechanisms of APT, DOM, HYS, and OND, as well as a probable anti-emetic mechanism of AA, based on their affinity for binding to the muscarinic, D_2_, D_3_, 5HT_3_, and NK_1_ receptors. In this case, AA acts as an inhibitor of D_2_, D_3_, 5HT_3_, M_4_, and NK_1_ receptors, while DOM, APT, OND, and HYS inhibit D_2_, NK_1_, 5HT_3_, and muscarinic receptors, respectively. The vomiting center (medulla oblongata) is kept from being triggered when these stomach receptors are blocked, preventing muscular contraction, GIT contraction, and the outcome of no emesis].

A synergistic effect was observed in this study by the combination drug therapy, which resulted in fewer retches and a longer latency period in chicks^67^. In our in vivo experiment, the combined group of (OND + AA-40) exhibited a significant percentage decrease in retches and an increase in latency period of 76.80% and 82.28%, respectively in comparison to the vehicle group. However, AA increases the antiemetic effect of DOM and HYS in the combination groups by showing a lower number of retching and elevated latency compared to the compound administered alone into the experimental animals. Depicted in Fig. 7 is the Suggested anti-emetic mechanism of the standard medications and test compound, AA.

The molecular docking approach attempts to predict the most effective orientation of a compound to its macromolecular target (receptor) when these molecules are bonded together to form an enduring complex^8,68^. Recently, computational investigations have made it possible to create, screen, and develop medication candidates in a novel way. This cuts down on expenses related to animals and laboratories as well as overall evaluation time^69^. Molecular affinity is employed to estimate the level of binding (interaction) between a ligand and a targeted protein^70^. Findings from our in silico study revealed that the test ligand AA exhibits comparatively higher affinity than the selected referral ligands against different types of receptors that are liable for stimulating emesis, such as H_1_, 5HT_3_, and various subtypes of muscarinic receptors (M_1_–M_5_). The M_4_ receptor, which is an essential part of the cholinergic system, can control the release of several neurotransmitters, including dopamine, in the brainstem’s CTZ, which evokes emesis^71^. Among several emesis-inducing muscarinic subtype receptors, the tested ligand (AA) exhibited the highest binding value (− 10.2 kcal/mol) against the M_4_ receptor and blocked the receptor activity. On the other hand, HYS yielded a binding score of − 8.9 kcal/mol toward the M_4_ receptor. Among several muscarinic subtypes’ receptors, M_3_ receptors are activated by acetylcholine, which is under systematic control. These receptors are abundant in smooth muscle and the GIT and are responsible for the contraction of the GI and gallbladder smooth muscles^72,73^. The M_4_ receptors are in the cortex and hippocampus among other parts of the brain, but they are most noticeable in the striatum, where it is hypothesized that they regulate dopamine production and locomotor activity^74,75^. The ligand (e.g., the neurotransmitter acetylcholine), which binds to the active site of the M_4_ receptor, starts the receptor activity. In this case, the number of amino acid residues that compose the binding site of the M_4_ receptor has not yet been fully identified, but this study has found some significant residues.

Based on our in silico study, and due to the binding of the tested ligand and reference medications with various receptors, numerous identical amino acid residues are formed, including THR433, VAL91, PHE410 for D_2_, VAL86, LEU89, VAL107, ILE183, PHE106 for D_3_, TYR404 for M_1_, TYR426 for M_2_, PHE186, TYR92, TRP435, TYR439 for M_4_, and HIS265, ILE113, PHE264, PHE268 for NK_1_. This signifies that they interact with the identically highlighted amino acid residues to form a coupling at the same area on the receptors. The highest docking score for the experimental ligand (AA) toward the M_4_ receptor is caused by the formation of one HB bond and multiple additional hydrophobic bonds. On the other hand, the standard drug HYS formed a lower number of hydrophobic bonds than the experimental ligand. Our findings also showed that the HB distance of AA is 2.24 Å, whereas it is 2.87 Å and 2.77 Å for HYS, which indicates that AA binds more closely to the receptor than HYS. Therefore, we anticipate that PHE186, TYR92, TYR439, and TRP435 are the key residues which implicated in the antagonizing action of AA against the M_4_ receptor. However, the solitary tract nucleus (STN) and the CTZ of the central nervous system have elevated concentrations of 5HT_3_ receptors^76^. It triggers nausea and vomiting by activating the appropriate emetic receptors on the vagal afferents^77^. The 5HT_3_ antagonists (e.g., ondansetron) prevent 5HT from activating both centrally in the CTZ and peripherally on GI vagal nerve terminals. This hindrance exerts potent antiemetic activity^78^. Our in silico investigation also revealed that AA exhibited a higher binding affinity against the 5HT_3_ receptor compared to the standard medication OND, the binding affinity is − 8.1 kcal/mol and − 6.9 kcal/mol, respectively. The tested ligand interacts with the 5HT_3_ receptor by forming one HB of ILE98 amino acid residue and several hydrophobic bonds with specific amino acid residues of PRO113, LYS25, PRO89, VAL95, TYR114 whereas, OND did not form any HB. Therefore, our findings show that AA exhibits potential antiemetic activity by blocking both the muscarinic and 5HT_3_ receptor pathways.

Drug-likeness is a fundamental guideline in the context of drug development and discovery, and it provides qualitative predictions about the probability that a chemical compound would be used in an oral medication in terms of sufficient bioavailability. It identifies the drug’s nature-related pharmacokinetics by assessing the drug’s physicochemical characteristics^8,22,79^. Lipinski’s rule of five is broadly used in predicting pharmacokinetics and drug-likeness. According to Lipinski’s rule of five, a drug candidate ought to have a MW of 500 g/mol or less, five or fewer HBD, ten or fewer HBA, and a lipophilicity (LogPo/w) of no more than five^80^. All ligands are predicted to have superior pharmacokinetic characteristics and are within the range of becoming medicines under Lipinski’s criterion. Our chosen test ligand meets each requirement of Lipinski’s rule of five and establishes improved pharmacokinetic characteristics.

For the development of secure and reasonably priced drugs, in silico toxicology studies are essential and critical^81^. Evaluating the effectiveness of possible medication candidates is the main goal of toxicology studies regarding the process of developing new drugs. The ultimate objective is to interpret animal responses to determine the risk to human subjects^82–84^. Toxicology testing is also crucial for determining any possible adverse effects that compounds may have. For instance, persistent chemical exposure in humans typically results in genotoxicity, immunotoxicity, carcinogenicity, and developmental and reproductive toxicity^85,86^. Results from this investigation showed that AA does not exhibit immunotoxicity, mutagenicity, carcinogenicity, or cytotoxicity-related toxic effects. However, it did show toxic effects in terms of hepatotoxicity. Due to its ability to antagonize muscarinic acetylcholine and 5HT_3_ receptors, our findings demonstrated that AA exhibits significant antiemetic activity against the CuSO_4_⋅5H_2_O-induced emesis. The synthetic antiemetics that are now on the market have been shown in several trials to exhibit a multitude of adverse effects, including diarrhea or constipation, lethargy, malaise, headache, visual changes, lightheadedness, and dry mouth^87,88^. In contrast, alternative antiemetic medications, particularly those made of natural ingredients, showed comparatively fewer adverse effects and effective therapeutic advantages^89,90^.

Studies utilizing specific laboratory animals give crucial information on the positive and negative effects of novel drug candidates as well as potential biopharmaceutical issues^91^. Consequently, each pre-clinical investigation supports medical researchers in assessing the potential of biologically active compounds for clinical trials. This study showed limitations such as a lack of clinical trials and results based on the behavioral representation of the animals. The probable antiemetic mechanism of AA in this study is based on the in silico and in vivo studies, and it does not present any actual antiemetic mechanism. Taken together, our findings revealed that AA exhibits a potent antiemetic effect in experimental animals by reducing the number of retching and elevating the latency of emesis. The in-silico investigation manifested the reasons behind the antiemetic effects of AA, possibly through the interaction of AA with 5HT_3_ and different subunits of muscarinic receptors.

## Conclusions

In conclusion, findings from this investigation indicated that AA exhibits remarkable dose-dependent anti-emetic activity with a diminishing retching of 11.66 ± 2.52 and an elevating latency period of 98.00 ± 2.44 s for 40 mg/kg in CuSO_4_⋅5H_2_O-induced emetic animals compared to the vehicle group of 66.83 ± 3.58 and 7.50 ± 0.92 s, respectively. On the other hand, the emetic symptoms were also notably attenuated in the experimental animals treated with the selected standards (DOM, HYS, and OND), but the efficacy of APT and DHM is comparatively low. In addition, findings from the in silico investigation show that AA successfully meets all the parameters of drug-likeness, and the molecular docking study revealed that the ligand AA has a greater binding affinity against muscarinic receptors, particularly the subtype M_4_ with a docking score of (− 10.2 kcal/mol) and 5HT_3_ with a docking score of (− 8.1 kcal/mol) compared to selected standards for these receptors, with docking scores of HYS (− 8.9 kcal/mol) and OND (− 6.9 kcal/mol) for M_4_ and 5HT_3_, respectively. Our results also showed that AA exhibits a synergistic effect when given with the selected referral drugs targeting various receptors liable for initiating emesis. The toxicological study also revealed that AA shows no toxic characteristics except hepatotoxicity. However, more investigations are suggested to identify the actual toxic mechanisms of AA. Furthermore, investigations are also required to establish a proper dose for humans through clinical trials and to investigate the exact mechanisms of action of AA in relieving vomiting and nausea brought on by several different reasons.

## Data Availability

The datasets used and/or analysed during the current study are available from the corresponding author on reasonable request.
